# Drug-resistant and hospital-associated *Enterococcus faecium* from wastewater, riverine estuary and anthropogenically impacted marine catchment basin

**DOI:** 10.1186/1471-2180-14-66

**Published:** 2014-03-14

**Authors:** Ewa Sadowy, Aneta Luczkiewicz

**Affiliations:** 1 National Medicines Institute, ul. Chelmska 30/34, Warsaw 00-725, Poland; 2 Gdansk University of Technology, ul. G. Narutowicza 11/12, Gdansk 80-952, Poland

**Keywords:** WWTP, Marine waters, Enterococcus, Resistance, HiRECC, Plasmid, Replicon type, Toxin-antitoxin system, Esp, Pili

## Abstract

**Background:**

Enterococci, ubiquitous colonizers of humans and other animals, play an increasingly important role in health-care associated infections (HAIs). It is believed that the recent evolution of two clinically relevant species, *Enterococcus faecalis* and *Enterococcus faecium* occurred in a big part in a hospital environment, leading to formation of high-risk enterococcal clonal complexes (HiRECCs), which combine multidrug resistance with increased pathogenicity and epidemicity. The aim of this study was to establish the species composition in wastewater, its marine recipient as well as a river estuary and to investigate the antimicrobial susceptibility of collected isolates. Molecular methods were additionally applied to test the presence of HiRRECC-related *E. faecium*.

**Results:**

Two wastewater treatment plants (WWTPs), their marine outfalls and Vistula river that influence significantly the quality of waters in Gulf of Gdansk were sampled to investigate the presence of *Enterococcus* spp. Four-hundred-twenty-eight isolates were obtained, including *E. faecium* (244 isolates, 57.0%), *E. hirae* (113 isolates, 26.4%) and *E. faecalis* (63 isolates, 14.7%); other species (*E. gallinarum/casseliflavus*, *E. durans* and *E. avium*) accounted for 1.9%. Antimicrobial susceptibility testing revealed the presence of isolates resistant to erythromycin, tetracycline, amipicillin, fluoroquinolones and aminoglycosides (high-level resistance), especially among *E. faecium*, where such isolates were usually characterized by multilocus sequence types associated with nosocomial lineages 17, 18 and 78 of this species representing HiRECC, formerly called CC17. These isolates not only carried several resistance determinants but were also enriched in genes encoding pathogenicity factors (Esp, pili) and genes associated with mobile genetic elements (MGE), a feature also typical for nosocomial HiRECC.

**Conclusions:**

Our data show that WWTPs constitute an important source of enterococcal strains carrying antimicrobial resistance determinants, often associated with the presence of MGE, for the recipient water environment, thus increasing a pool of such genes for other organisms. The presence of HiRECCs in wastewaters and marine/river environment may indicate that adaptations gained in hospitals may be also beneficial for survival of such clones in other settings. There is an obvious need to monitor the release and spread of such strains in order to elucidate better ways to curb their dissemination.

## Background

Enterococci are commensal bacteria of intestinal tract of mammals and birds, but these bacteria can also be found in the gut of reptiles and insects, and in different environmental compartments like soil and plants, in food (both as starters and contamination) as well as in aquatic ecosystems, both distributed with treated wastewater and lacking such anthropogenic impact [1]. In the human intestine, the *Enterococcus faecalis* and *Enterococcus faecium* are most abundant, and these two species are also most commonly isolated from human enterococcal infections, however, other species, such as *Enterococcus avium*, *Enterococcus casseliflavus*, *Enterococcus durans*, *Enterococcus gallinarum*, and *Enterococcus raffinosus* have also been detected [2,3]. Recently *E. faecalis* and *E. faecium* play more and more important role in hospital-acquired infections (HAIs), such as bacteremia, endocarditis, infections of urinary tract and post-surgery wounds [4], and nowadays represent a second etiologic agent of hospital bloodstream infections in Europe [5]. In the recent years, the relative proportion of *E. faecium* to *E. faecalis* is increasing in the US and Europe [6-8], which is likely due to the spread of a particular hospital-adapted polyclonal high-risk enterococcal complex (HiRECC) of *E. faecium*, initially described as clonal complex 17, CC17 [8]. This nosocomial subpopulation consists of three main lineages, named from the central sequence type (ST), defined by multilocus sequence typing (MLST), as lineages 17, 18 and 78 [9]. The acquisition of resistance to antimicrobials of several classes constitutes an important feature of hospital-associated *E. faecium,* what not only renders them resilient to therapy but also creates a reservoir of antimicrobial resistance genes, often associated with mobile genetic elements (MGE), such as conjugative transposons and plasmids [10]. The most important from the clinical point of view examples of acquired resistance among enterococci include resistance to (amino)penicillins, fluoroquinolones, glycopeptides (vancomycin-resistant enterococci, VRE) and high-level resistance to aminoglycosides (HLAR) [3]. Resistance to ampicillin, associated with the changes in penicillin binding protein 5 (PBP5) and resistance to quinolones, determined by point mutations in genes encoding bacterial gyrase and topoisomerase IV are characteristic phenotypic features of nosocomial *E. faecium*[11,12]. Such strains also commonly carry resistance genes to other classes of antimicrobials, such as tetracyclines {*tet*(M), *tet*(L), *tet*(S) and *tet*(O)}, macrolides {*erm*(B)} and aminoglycosides [13]. The HLAR phenotype is usually determined by the *aac(6′)-Ie-aph(2″)* gene, encoding a so-called bi-functional enzyme responsible for resistance to all aminoglycosides, except for streptomycin; high-level resistance to this compound is typically specified by *ant(6′)-Ia*[3]. Hospital-associated *E. faecium* is also enriched in several virulence factors, such as enterococcal surface protein (Esp) and MSCRAMM proteins [14-16].

Several studies have addressed the spread of HiRECCs, especially VRE, within as well as among health-care facilities [17]. Much less is known, however, about the release of drug-resistant enterococci and HiRECCs into the environment, and their subsequent survival. Since it is suspected that human-associated bacteria can be regarded as vectors of gene transmission into environmental populations [18] and positive selection of bacteria resistant to antimicrobial agents was observed in wastewater processes [19,20], the potential role of effluents from wastewater treatment plant (WWTP) in dissemination of clinically relevant bacteria and genes needs evaluation. It is particularly important in ecosystems subjected to strong anthropogenic impact, such as the Gulf of Gdansk and its shallow western part, the Puck Bay. Significant pollution load is discharged to this coastal area through the numerous local rivers and marine outfalls. Due to limited water exchange in the Gulf of Gdansk, safe wastewater disposal is essential to prevent the environmental degradation and to preserve the public health. Epidemiological studies reported a direct relationship between the risk of gastroenteritis among swimmers and density of enterococci in surface waters [21]. Additionally, resistant and multiresistant enterococci were detected in effluent of WWTPs [18,20,22], suggesting their survival capacity in treatment processes. However, no microbiological standards have been set on WWTP effluents in the majority of European countries and it is required instead to monitor the quality of recreational water [23]. The US Environmental Protection Agency [21] as well as the European Union legislation [23] recommended detection of fecal enterococci, together with *Escherichia coli* as indicators of fecal contamination of bathing water.

**Figure 1:**
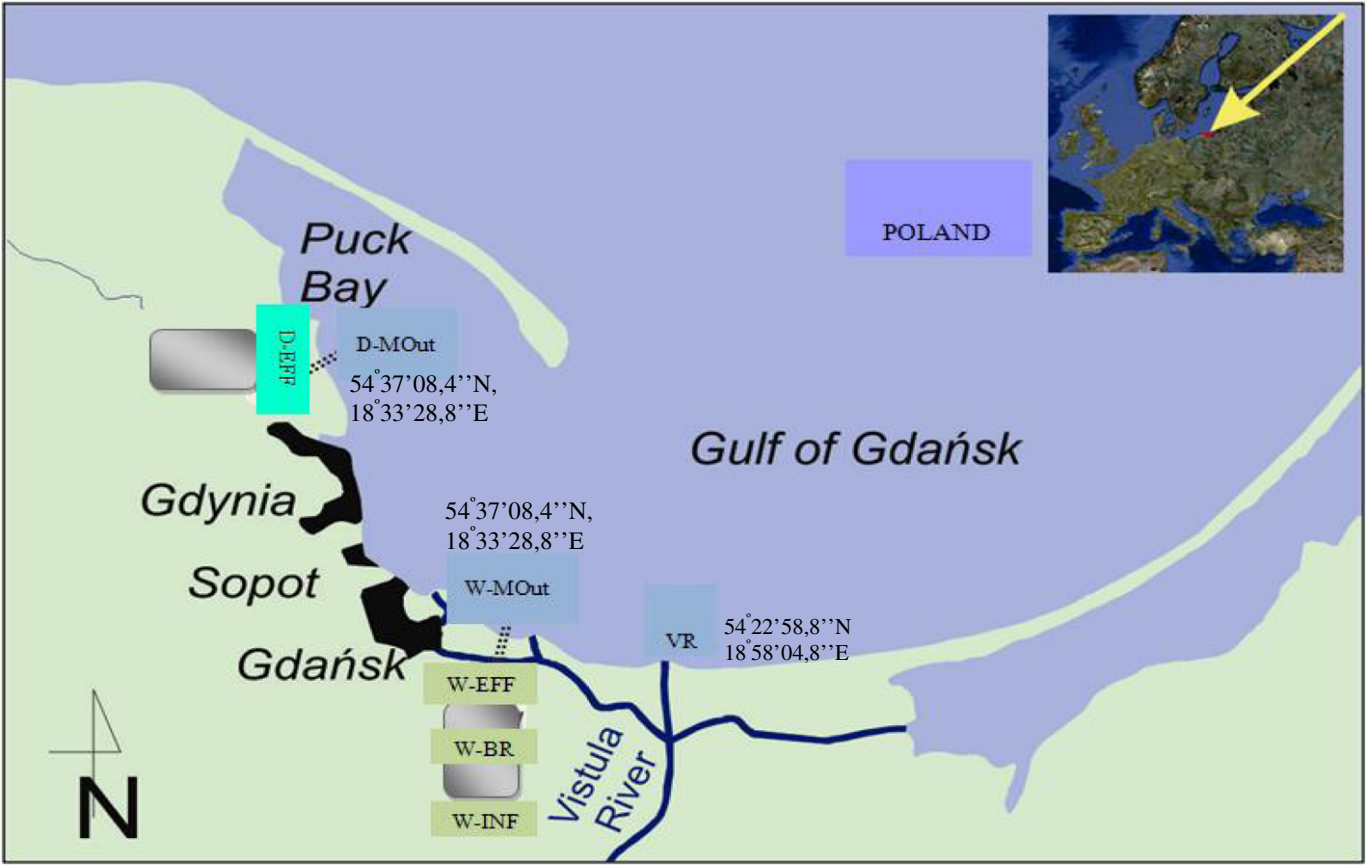
**Sampling sites location: WWTP Gdansk-Wschod – influent (W-INF), bioreactor (W-BR), effluent (W-EFF) and marine outfall (W-MOut); WWTP Gdynia-Debogorze effluent (D-EFF) and marine outfall (D-MOut); Vistula River mouth (VR).** Both WWTPs treat mainly municipal wastewater; industrial and not disinfected hospital wastewater consist approximately 10% and 0.2% of daily inflow, respectively. The WWTP Gdansk-Wschod works in a modified University of Cape Town system; Qav. = 96 000 m^3^/d; serves 570 000 people; in operation since 2001. The WWTP Gdynia-Debogorze works in Bardenpho system, including the Barnard modification; Qav. = 55 000 m^3^/d; serves 350 000 people; in operation since 2011. The Vistula River is the second biggest river (1 048 km long) in the catchment’s area of the Baltic Sea; the average flow at the mouth to the Baltic Sea ca. 1050 m^3^·s^−1^.

Aim of the current study was to investigate the presence and species composition of enterococci in treated wastewater, river estuary water and their recipient, highly anthropogenically impacted Gulf of Gdansk. For this purpose, enterococci were isolated from wastewater of two local WWTPs, Gdansk-Wschod (influent - W-INF, effluent - W-EFF and bioreactor - W-BR) and Gdynia-Debogorze (effluent - D-EFF) as well as from their marine outfalls (W-MOut and D-MOut, respectively). Additionally, the Vistula River mouth was sampled, due to ecological importance of this river flows for the Gulf of Gdansk area (Figure 1). Identification and further characterization of drug-resistant enterococci, especially the most prevalent *E. faecium*, including its resistance, virulence and MGE genes was in the special focus of the study, due to the problem posed by such clones for safe, economical and reliable way of wastewater disposal in costal ecosystems.

## Results

### Species composition and antimicrobial susceptibility of enterococcal isolates

In this study, the number of enterococci in WWTP effluents (W-EFF and D-EFF) remained high for both WWTP, yielding up to 6.1x10^5^ CFU per 100 mL (Table 1), although the WWTP Gdansk-Wschod showed over 99% efficiency in the removal of enterococci. In marine outfalls of the two WWTPs (W-MOut, D-MOut) as well as at the Vistula River mouth, enterococci were detected at low densities, i.e. below 100 CFU per 100 mL. Altogether, 428 isolates were collected, including 243 isolates from wastewater, 116 isolates from marine outfalls, and 69 from Vistula River mouth (Table 1). These 428 isolates were mainly identified as *E. faecium* (altogether 244 isolates, 57%), *Enterococcus hirae* (113 isolates, 26.4%) and *E. faecalis* (63 isolates, 14.7%), with an occasional occurrence of *E. gallinarum/casseliflavus, E. durans* and *E. avium* (4, 3, and 1 isolates, respectively, altogether 1.9%). The most common species in all sampled sites was *E. faecium* (56.2% – 65.5%), with the exception of Vistula River mouth where *E. hirae* was predominant (53.6% of isolates), followed by *E. faecalis* (37.7%).

**Table 1:** **Presence (CFU 100 mL**^**−1**^**) of enterococci, number (%) of enterococcal isolates recovered and species composition at particular sites**

		**WWTP**						**Vistula river**	**Total**
		**Gdansk-Wschod**				**Gdynia-Debogorze**			
		**W-INF**	**W-BR**	**W-EFF**	**W-MOut**	**D-EFF**	**D-MOut**	**VR**	
Presence	CFU 100 mL^−1^	(0.7–23) × 10^7^	*na*	(0.1–6.1) × 10^5^	3 – 60	(0.8–3.1) × 10^5^	2 – 80	1 – 30	-
Number (%) of recovered isolates		33 (7.7)	55 (12.8)	82 (19.2)	45 (10.5)	73 (17.1)	71 (16.6)	69 (16.1)	428 (100)
Species composition: number (%)	*E. faecium*	19 (57.6)	36 (65.5)	53 (64.6)	29 (64.4)	41 (56.2)	40 (56.3)	26 (37.7)	244 (57)
	*E. faecalis*	5 (15.2)	8 (14.5)	19 (23.2)	4 (8.9)	19 (26.0)	3 (4.3)	5 (7.2)	63 (14.7)
	*E. hirae*	8 (24.2)	11 (20.0)	7 (8.5)	11 (24.4)	12 (16.4)	27 (38.0)	37 (53.6)	113 (26.4)
	*E.casseliflavus*/*gallinarum*	1 (3.0)	*nd*	2 (2.5)	*nd*	1 (1.4)	*nd*	*nd*	4 (1.0)
	*E. durans*	*nd*	*nd*	1 (1.2)	1 (2.3)	*nd*	1 (1.4)	*nd*	3 (0.7)
	*E. avium*	*nd*	*nd*	*nd*	*nd*	*nd*	*nd*	1 (1.5)	1 (0.2)

*na* – not applicable, *nd* – not detected.

Abbreviations of sampling points’ locations and their description provided in Figure 1.

In the next step, antimicrobial susceptibility was tested for *E. faecium* and *E. faecalis* isolates (Table 2). It should be noted that at some points a low number of *E. faecalis* isolates was recovered (fewer than 10 isolates) and thus, certain resistance rates obtained for *E. feacalis* should be treated with caution. Resistance to erythromycin was detected with the highest prevalence among both species, however, rates of resistance to this compound varied over a broad range. Erythromycin-resistant *E. faecium* comprised 36.8% of isolates in raw wastewater (W-INF), 45.3% in treated wastewater (W-EFF) and 58.6% in marine outflow (W-MOut) of WWTP Gdansk-Wschod. Similar tendency was seen for *E. faecalis* isolates where resistance rates in the sampling points listed above were equal to 20.0%, 68.4% and 100%, respectively. For WWTP Gdynia-Debogorze effluent (D-EFF) and marine outflow (D-MOut), erythromycin resistance rates for *E. faecium* varied between 68.2% and 55.0%, respectively, while for *E. faecalis* were equal 68.4% and 66.7%, respectively. In the case of fluoroquinolones, resistance to ciprofloxacin among *E. faecium* varied between 30.8% in Vistula River mouth to 44.8% in W-MOut, while resistance to levofloxacin did not exceed 20%. Among *E. faecalis* resistance to ciprofloxacin and levofloxacin was, in general lower then reported for *E. faecium* in the corresponding sampling points (up to 25.0%), except Vistula River mouth. The high-level streptomycin resistance (HLSR) was prevalent and reached 20.7% for *E. faecium* isolates from marine outflow of WWTP Gdansk-Wschod (W-MOut). Also for *E. faecalis*, HLSR phenotype was detected among up to 16% of all tested isolates. Resistance to glycopeptides, linezolid and daptomycin was not detected among all tested enterococci.

**Table 2:** **Antimicrobial resistance of** ***E. faecium*** **and** ***E. faecalis***

	**Number (%) of isolates**									
	**Resistant to a single antimicrobial agent**								**Sensitive**	**MDR phenotype**
	**GM**	**ST**	**AM**	**CIP**	**LVX**	**TE**	**SYN**	**E**	**S**	
***E. faecium n = 244***										
W = INF (n = 19)	1 (5.3)	3 (15.8)	1 (5.3)	6 (31.6)	1 (5.3)	5 (26.3)	6 (31.6)	7 (36.8)	6 (31.6)	4 (21.1)
W-BR (n = 36)	1 (2.8)	2 (5.6)	1 (2.8)	12 (33.2)	3 (8.3)	7 (19.4)	19 (52.8)	16 (44.4)	10 (27.8)	8 (22.2)
W-EFF (n = 53)	3 (5.7)	2 (3.8)	4 (7.5)	23 (43.4)	10 (18.9)	10 (18.9)	18 (34.0)	24 (45.3)	12 (22.6)	14 (26.4)
W-MOut (n = 29)	0	6 (20.7)	6 (20.7)	13 (44.8)	4 (13.8)	8 (27.6)	14 (48.3)	17 (58.6)	5 (17.4)	9 (31.0)
D-EFF (n = 41)	1 (2.4)	6 (14.6)	5 (12.2)	13 (31.7)	2 (4.9)	10 (24.4)	12 (29.2)	28 (68.2)	4 (9.8)	8 (19.5)
D-MOut (n = 40)	1 (2.5)	2 (5)	2 (5)	14 (35.0)	3 (7.5)	2 (5.0)	21 (52.5)	22 (55.0)	7 (17.5)	3 (7.5)
VR (n = 26)	0	1 (3.8)	0	8 (30.8)	1 (3.8)	2 (7.7)	15 (57.7)	11 (42.4)	3 (11.5)	3 (11.5)
All (n = 244)	7 (2.9)	22 (9.0)	19 (7.8)	90 (36.9)	24 (9.8)	44 (18.0)	105 (43.0)	125 (51.2)	49 (20.1)	47 (19.3)
***E. faecalis n = 63***										
W-INF (n = 5)	*0*	1 (20.0)	0	1 (20.0)	0	1 (20.0)	*na*	1 (20.0)	4 (80.0)	0
W-BR (n = 8)	2 (25.0)	2 (25.0)	1 (12.5)	2 (25.0)	2 (25.0)	2 (25.0)	*na*	7 (87.5)	1 (12.5)	2 (25.0)
W-EFF (n = 19)	0	1 (5.3)	1 (5.3)	3 (15.8)	3 (15.8)	4 (21.1)	*na*	13 (68.4)	0	3 (15.8)
W-MOut (n = 4)	0	0	0	0	0	0	*na*	4 (100)	0	0
D-EFF (n = 19)	2 (10.5)	5 (26.3)	0	1 (5.3)	0	9 (47.4)	*na*	13 (68.4)	4 (21.1)	5 (26.3)
D-MOut (n = 3)	1 (33.3)	1 (33.3)	0	0	0	2 (66.7)	*na*	2 (66.7)	0	1 (33.3)
VR (n = 5)	0	0	0	2 (40.0)	0	0	*na*	1 (20.0)	2 (40.0)	0
All (n = 63)	5 (7.9)	10 (15.9)	2 (3.2)	9 (14.3)	5 (7.9)	18 (28.6)	*na*	41 (65.1)	11 (17.5)	11 (17.5)

GM, gentamicin; ST, streptomycin; AM, ampicillin; CIP, ciprofloxacin; LVX, levofloxacin; TE, tetracycline; SYN, quinupristin-dalfopristin; E, erythromycin; S, sensitive to all tested antimicrobial agents; MDR, multiple drug resistance; *na,* not applicable.

Abbreviations of sampling points’ locations and their description provided in Figure 1.

### Clonal structure of *E. faecium* isolates, and distribution of virulence, resistance and MGE genes

**Figure 2:**
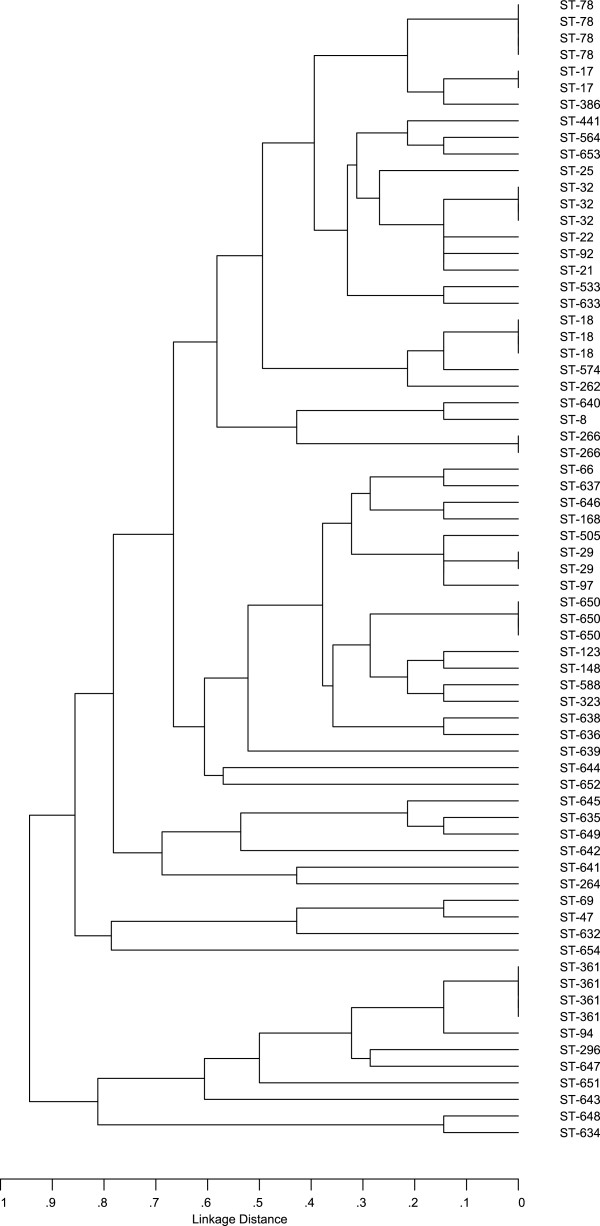
**MLST-based similarity tree of** ***E. faecium*** **isolates, constructed using the START software and the UPGMA clustering algorithm.**

**Figure 3:**
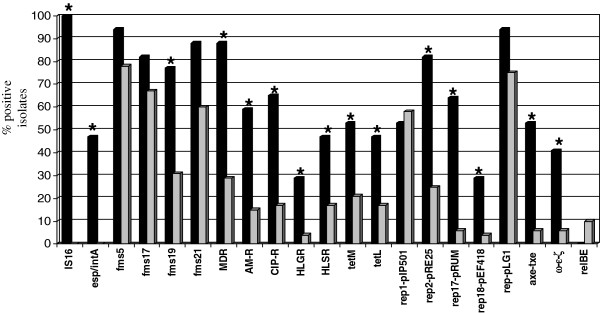
**Distribution (%) of IS*16*, resistance phenotypes and virulence, resistance and plasmid genes among*E. faecium*isolates.** Black columns, isolates with STs typical for nosocomial *E. faecium,* grey columns, other *E. faecium* isolates. Significant differences (p < 0.05) between two groups are indicated by an asterisk. MDR, multi-drug resistance; AM-R, ampicillin resistance; CIP-R, ciprofloxacin resistance; HLGR, high-level gentamicin resistance; HLSR, high-level streptomycin resistance.

Sixty-nine isolates of *E. faecium* (28.3% of all isolates of this species) were further subjected to MLST and analysis of the presence of resistance, virulence and MGE genes. These isolates presented various phenotypes of resistance to antimicrobial compounds, as described below, and were obtained mostly from effluents and marine outfalls of both WWTPs (Table 3). MLST discerned 54 different STs (Figure 2), including 23 new ones (STs 632–654). The comparative eBURST analysis against the whole MLST *E. faecium* database (as of the 5^th^ April 2013) grouped the 38 STs, specific for 50 isolates into the major ‘super-complex 17’, characteristic for this species (data not shown). Further detailed analysis of the localization of particular STs within this structure revealed that 11 STs characteristic for 17 isolates belonged to the nosocomial complex of *E. faecium,* representing all three major lineages 17, 18 and 78 of a former CC17, as defined by Willems *et al.*[9] (Table 3). Seventeen STs (20 isolates), nine STs (11 isolates) and two STs with single isolates each belonged to CC5, CC22 and CC9, respectively, as defined by Freitas *et al.*[24]. Of the remaining 16 STs, nine were included into six small CCs (CCs 27, 46, 94, 296, 639, 648), unlinked to the major ‘super-complex’; ST632 formed a ‘doubleton’ (i.e. was linked by a single-locus variant link to only one ST from the database) and six STs, represented by single isolates, were singletons, i.e. were not related to any ST in the database at the time of analysis. Thirty isolates (43.5%) in the investigated group showed the MDR phenotype, which was significantly associated (p = 0.00006) with isolates from the nosocomial complex (Table 3 and Figure 3) but found also in CC5, CC22 and CC94; this phenotype did not occur among small CCs or singletons. Seventeen isolates, representing the nosocomial complex were additionally studied by multilocus VNTR (variable number tandem repeats) analysis (MLVA) to better elucidate the relationships among these isolates. Altogether, nine MLVA-types (MTs) were found (Table 3). Importantly, the same MLVA-type, MT159 associated with ST78 was characteristic for isolates derived from samples from W-EFF and W-MOut, suggesting survival of this particular clone.

**Table 3:** **Distribution of virulence genes and resistance determinants among lineages, CCs and STs of** ***E. faecium***

**Lineage/CC/ST**	**MT**	**Isolation site**	**Virulence genes**					**Resistance determinants**								**Transposon genes**		**Plasmid** ***rep*** **genes**						**Plasmid TAS**		
			***esp/intA***	***fms5***	***fms17***	***fms19***	***fms21***	***pbp5***	**GyrA**	**ParC**	***aac(6′)-Ie-aph(2″*)**	***ant(6′)-Ia***	***tet*** **(M)**	***tet*** **(L)**	***tet*** **(S)**	***intTn916***	***tndX***	***rep1***_**pIP501**_	***rep2***_**pRE25**_	***rep17***_**pRUM**_	***rep18***_**pEF418**_	***rep***_**pLG1**_	***rep***_**pMG1**_	***axe-txe***	**ω** ***-ε-ζ***	***relBE***
Lineage 17																										
**17** (2)	1, 8	W-EFF	2	2	2	2	2	*pbp5-30*	S84R	R61G S80R			1	1				2	2	1	2	2		1	1	
**386**	7	W-MOut	1	1	1	1	1					1						1	1	1	1	1		1	1	
Lineage 18																										
**18** (3)	1 (3)	D-EFF, W-MOut		3	3	3	1	*pbp5-20*	S84Y	R61G S80I	1	2	3	3				1	2	1		3				
**262**	10	W-EFF		1	1		1	*pbp5-25*	S84Y	R61G	1		1	1				1	1			1			1	
574	402	VR		1			1									1						1				
Lineage 78																										
**78** (4)	159 (4)	W-EFF, W-MOut	4	4	4	4	4	*pbp5-28*	S84R/Y	R61G S80R	2	1	2	2				1	4	4	2	4		4	3	
**266** (2)	139 (2)	W-INF, D-EFF		2	1	2	2	*pbp5-24*				1	1			2		1	1	2		2		2		
**323**	12	D-MOut		1	1	1	1	*pbp5-26*	*wt*	R61G S80I	1	1	1	1			1	1	1			1				
**564**	302	D-EFF	1	1	1		1	*pbp5-29*	S84I	R61G S80R		1						1	1	1		1		1	1	
**653**	302	W-EFF					1	*nd*	S84I	R61G S80R		1							1	1						
CC5																										
29 (2)		W-EFF, W-MOut		2	1		1											2				1				
**66**		W-MOut		1	1		1						1	1								1		1		
97		W-EFF					1		*wt*	R61G S80R								1				1				
123		W-EFF		1		1			*wt*	R61G								1			1	1				
**148**		W-MOut		1				*pbp5-30*	*wt*	R61G S80R		1	1	1			1					1				
**168**		W-MOut		1	1			*pbp5-31*				1	1	1		1		1	1	1		1		1		
505		W-EFF		1					*wt*	R61G								1				1				
**588**		D-MOut		1				*pbp5-30*				1	1	1			1		1			1				
635		D-EFF			1		1											1				1				
636		W-EFF		1					*wt*	R61G								1								
637		W-EFF		1	1		1		*wt*	R61G								1				1				
638		W-EFF			1				*wt*	R61G S80I								1								
642		W-EFF		1	1	1												1				1				
645		D-EFF		1	1													1				1				1
**646**		W-EFF		1	1								1			1		1								
**649**		W-BR		1	1	1		*pbp5-32*	*wt*	R61G		1														
**650** (3)		D-EFF		3	3			*pbp5-27*				2	2	2		1		1	3							
CC 9																										
8		D-EFF		1	1	1	1					1	1						1	1	1	1		1		
**640**		W-EFF		1	1	1	1															1				
CC 22																										
**21**		W-EFF			1	1	1					1	1	1		1						1				
22		W-EFF		1	1		1									1	1					1				
**25**		W-EFF			1		1	*pbp5-32*	*wt*	R61G	1								1			1				
32 (3)		W-EFF		3	3	1			*wt*	R61G								2				1				
92		W-EFF		1	1		1											1				1				
441		W-EFF		1	1		1						1	1					1			1			1	
**533**		W-EFF		1																		1	1			
**633**		W-EFF		1									1		1	1		1								
644		D-EFF		1	1		1											1				1				
CC27																										
652		W-EFF			1		1											1				1				
CC46																										
47		VR		1			1											1				1				
69		D-EFF		1	1		1											1	1			1				
CC94																										
94		W-MOut			1		1											1				1				
**361** (4)		D-EFF, D-MOut		3	4	4	4	*pbp5-29*				1		1						1		4				1
CC296																										
296		W-EFF		1		1																				
CC639																										
639		W-EFF		1	1		1											1				1				
CC648																										
634		W-EFF		1			1											1				1				1
648		W-EFF		1	1		1											1				1				
Doubletons																										
632		W-MOut		1			1															1				
Singletons																										
264		W-INF		1		1	1											1	1			1			1	
641		D-EFF		1	1		1															1				
643		W-EFF		1			1											1	1			1			1	
647		W-MOut				1	1											1				1	1			
651		D-EFF			1	1	1												1			1				1
654		VR				1	1											1	1			1				1
**All (69)**			**8**	**57**	**49**	**29**	**46**				**6**	**17**	**20**	**17**	**1**	**9**	**4**	**39**	**27**	**14**	**7**	**55**	**2**	**12**	**10**	**5**

CC, clonal complex; ST, sequence type; MT, MLVA-type; *nd*, not determined due to lack of amplification of the *pbp5* gene; *wt*, wild-type; STs associated with MDR phenotype in bold**.**

Abbreviations of sampling points’ locations and their description provided in Figure 1.

IS*16* was present in all 17 isolates included by MLST to three lineages of nosocomial *E. faecium* and this marker was limited to this group (Table 3 and Figure 3). The *esp*_Efm_ gene occurred always together with *intA* among all isolates of lineage 17 and in some isolates of lineage 78. The *fms19* pilin gene*,* detected in 29 isolates, was over-represented among STs typical for nosocomial *E. faecium* while other pilin genes, such as *fms5, fms17* and *fms21,* detected in 57, 49 and 46 isolates, respectively, occurred among various CCs and singleton STs. Ampicillin resistance, significantly associated with isolates of nosocomial complex (10 of 17 isolates, 58.8%; p = 0.001), occurred also in CC5 (6 isolates), CC22 and CC94 (1 isolate each). Sequencing of the 3′ part of the *pbp5* gene revealed its 11 nucleotide alleles corresponding to 11 variants of the C-terminal part of PBP5. Six alleles of the gene were new (*pbp5-24* to *pbp5-29*), two alleles (*pbp5-20, pbp5-22*) were described previously among Polish hospital VREm isolates [13] and three alleles were found in other studies: *pbp5-30* occurred in a Portuguese animal strain of *E. faecium*[25] and *pbp5-31* and *pbp5-32* in clinical isolates TCGE70411_2 and 164306 also from Portugal (deposited as JN208888 and JN208885 in the GenBank, respectively). For a single ampicillin-resistant isolate of ST653 (lineage 78), *pbp5* amplification was not achieved despite repeated attempts. The majority (11 of 17 isolates, 64.7%) of isolates belonging to three lineages of nosocomial *E. faecium* showed resistance to ciprofloxacin determined by mutations R61G and S80I/R in ParC and S84I/R/Y in GyrA. Ciprofloxacin resistance was also significantly over-represented among isolates of STs typical for nosocomial *E. faecium* (p = 0.0006). Nine isolates resistant to ciprofloxacin were found also in CCs 5, 9 and 22. These isolates possessed mutations in ParC mentioned above but had the wild-type QRDR in GyrA. HLGR occurred in five isolates of lineages 18 and 78, and in single isolates of CC5 and CC22; this phenotype was determined by the presence of *aac(6′)-Ie-aph(2″*) with an exception of an isolate from CC5 which was negative for this gene. High-level resistance to streptomycin (HLSR) was widely distributed among studied isolates, and determined by the *ant(6′)-Ia* gene. Tetracycline resistance was found also in various STs; the major determinant of this phenotype was *tet*(M) (20 isolates), followed by *tet*(L) (17 isolates). These two genes occurred together in 16 isolates. A single isolate of ST633, belonging to CC22, harbored *tet*(S)*.* The *tet*(O) gene was absent in the studied group. For a single tetracycline-resistant isolate of ST17 none of the four tetracycline determinants studied was detected. Nine isolates carried *int* of Tn*916*, and four the *tndX* gene of Tn*5397.* These transposon-specific genes were not characteristic for any lineage or complex (Table 3). Plasmid-replication genes such as *rep2*_pRE25_*, rep17*_pRUM_ and *rep18*_pEF418_ (26, 14 and 7 isolates, respectively), and genes specifying toxin-antitoxin systems (TAS), *axe-txe* and ω*-ε-ζ* (12 and 10 isolates, respectively) were significantly associated with the group of isolates of nosocomial *E. faecium.* Two other *rep* genes (*rep1*_pIP501_*, rep*_pLG1_) were widely distributed among all studied *E. faecium* isolates and the *rep*_pMG1_ gene occurred in two isolates (in a single isolate of ST533 from CC22 and in a singleton ST647). All ω*-ε-ζ*-positive isolates carried *rep2*_pRE25_*,* and nine isolates showed a concomitant presence of *axe-txe* and *rep17*_pRUM_. The *relBE* genes were found solely among five non-nosocomial *E. faecium* (a difference in distribution without statistical significance, p = 0.43), among various STs.

## Discussion

In this study, *Enterococcus* spp. was used to evaluate the impact posed by treated wastewater of two local WWTPs on their receiver, the costal waters of Gulf of Gdansk. Total counts of enterococci observed for raw (W-INF) and treated (W-EFF, D-EFF) wastewater are consistent with our previous study, showing similar, over 99% reduction capacity of applied treatment processes. It is also reported in the literature that wastewater treatment processes based on activated sludge, although they focus on removal of nutrients and organic matter, simultaneously reduce faecal indicators with high efficiency, reaching up to 99.9% [18,22]. Due to the high number of enterococci in raw wastewater, they are nevertheless released from a WWTP in a number up to 6.1x10^5^ CFU per 100 mL. Thus, marine water sampled in the area directly impacted by treated wastewater (W-MOut and D-MOut) showed a higher number of enterococci than the one observed in Vistula River mouth, however, lower by four orders of magnitude in comparison to corresponding treated wastewater (W-EFF and D-EFF). It should be noted that in marine outfalls (W-MOut, D-MOut) as well as in Vistula River mouth enterococci were detected in number lower than 100 CFU per 100 mL, i.e. the value set out by New Bathing Water Directive 2006/7/EC for the costal water of “excellent quality” [23].

It is suggested that enterococcal species composition in faecal-impacted environment depends from the source of contamination. In this study, two species most commonly colonizing humans, *E. faecalis* and *E. faecium* comprised together from 73% to 88% of enterococcal isolates in tested wastewater samples (W-INF, W-BR, W-EFF and D-EFF), 73% and 61% in marine outflows (W-MOut and D-MOut, respectively) and 45% in the Vistula River mouth. The predominant species in wastewater and marine water impacted by treated wastewater was *E. faecium* (from 56 to 65%), which stayed at a similar level during treatment processes, as determined also in our previous study [20]. Species distribution in wastewater samples is not well understood, and affecting factors may include differences in diet, climate, season and methods of detection. *E. feacalis* was found to be the most prevalent species in wastewater samples in Sweden [26], *E. faecium* in Spain, UK, Canada, France and Switzerland [26-29], while *E. hirae* predominated in Portugal and United States [22,30]. Although *E. faecalis* is typically the most abundant colonizer of humans in the community (e.g. [26,31]), *E. faecium* is generally most often associated with human microbial contamination [27,29]. *E. faecalis* was reported from agriculture-impacted waters [32] and *E. hirae* likely originates mainly from cattle and pig faecal contamination [26], which explains high relative proportion of these two species in Vistula River mouth. Apart from the variability of contamination sources, proportion of particular species may differ due to the differences of ability to sustain environmental stresses. In this study, enterococcal composition might have been influenced by wastewater treatment processes and marine water characteristics.

The effect of wastewater treatment and marine environment was seen on phenotypes of antimicrobial resistance. The positive selection of bacteria with resistance patterns has been already suggested and noted in wastewater processes [19,20,22]. In this study, the resistance rates to ampicillin, ciprofloxacin, levofloxacin and erythromycin, noted for *E. faecium* from raw wastewater (W-INF) were lower than these observed for this species in the corresponding treated wastewater (W-EFF), however, not statistically significant (p > 0.05). Moreover, resistance rates to ampicillin, ciprofloxacin, tetracycline and erythromycin observed in marine outflow of WWTP Gdansk-Wschod (W-MOut) were higher than in treated wastewater of this plant. Such phenomenon was not observed for treated wastewater of WWTP Gdynia-Debogorze (D-EFF) and its marine outfall (D-MOut). Resistance rates among *E. faecium* originating from D-MOut were, in general, comparable to these detected in the Vistula River mouth. It should be noted that treated wastewater from WWTP Gdansk-Wschod has been discharged via marine outfall (W-MOut) for last 12 years while marine outfall of WWTP Gdynia-Debogorze (D-MOut) has been operating for last two years. The observed differences in resistance rate corresponding to the local impact caused by long and short-term operated marine outfalls (W-MOut and D-MOut) need further attention.

Significant rates of antimicrobial resistance, observed for isolates of *E. faecalis* from wastewater and marine/river waters, impacted by wastewater, are in agreement with a recent study performed in six European countries, showing that in Poland the rates of resistance for human isolates of this species were usually among the highest, both in the community and hospitals [33]. To our knowledge, no corresponding data are available for *E. faecium* colonization in the community in Poland but a study focused on hospital VRE isolates showed their ubiquitous MDR phenotype and a high load of resistance genes [13]. In our study no vancomycin resistance were detected, likely due to relatively low (below 10%) prevalence of VRE in Polish hospitals [5], although such isolates are isolated from the environment in other countries e.g., [34].

This study shows that hospital clones of *E. faecium* are important carriers of resistance genes in wastewaters. Most likely, in a significant part they originated from hospital wastewater treated together with communal wastewater. In our estimation, hospital wastewater constitutes approximately 0.2% of both WWTPs’ daily inflow. The release of hospital-associated clones of *E. faecium* to the environment was observed also by others [24,28,35,36]. In our study, isolates belonging to the major nosocomial HiRECC (formerly named CC17) constituted altogether 24.6% of all isolates of *E. faecium* subjected to typing, and were present at all sampling sites. These isolates, apart from their inclusion into the HiRECC by MLST, demonstrated several other phenotypic and genotypic features typical for nosocomial *E. faecium,* i.e. they were resistant to ciprofloxacin and ampicillin, in vast majority showed MDR phenotype, and carried several resistance determinants, associated both with mutations of chromosomal genes (*gyrA, parC, pbp5*) and, in the case of aminoglycoside and tetracycline resistance, with gene acquisition. Plasmids likely played an important role in the later process, as some plasmid genes, such as *rep2*_pRE25_*, rep17*_pRUM_ and *rep18*_pEF418_, and TAS genes *axe-txe* and ω*-ε-ζ* were significantly more abundant in isolates associated with HiRECC than among the remaining isolates. As yet, the knowledge of distribution of plasmid-associated genes in populations of *E. faecium,* especially in the context of their clonal composition, remains limited. A study on 93 isolates of different geographical origins and from various sites showed an over-representation of *rep17*_pRUM_ and *rep*_pMG1_ among nosocomial *E. faecium,* while, in contrast to our observations, *rep18*_pEF418_ was not detected [37]. Moreover, we did not observe *rep*_pMG1_ among HiRECC of *E. faecium.* These differences may be due to a high prevalence of VRE (58 isolates) among 93 isolates studied by Rosvoll *et al*. what may explain differences in the plasmid content. The same study also reported the co-localization of ω*-ε-ζ* with *rep2*_pRE25_ and *axe-txe* with *rep17*_pRUM_ on the same plasmids, in concordance with our findings, showing a frequent joint occurrence of these pairs of genes. Another study, performed on 99 invasive isolates from Norway, mostly of HLGR phenotype, showed a significantly higher prevalence of *rep2*_pRE25_ and *rep17*_pRUM_ among the major nosocomial HiRECC [38]. This study as well some others [24,39] also demonstrated a common presence of *rep*_pLG1_ among *E. faecium* from hospital settings and in animals*.* Our findings indicate that this type of replicon is even more ubiquitous in *E. faecium* than previously reported*.* All *rep* genes mentioned above are typically associated with plasmids carrying various antimicrobial resistance genes [39,40], GenBank accession number AF408195. In addition to genes determining antimicrobial resistance and genes associated with MGE, isolates of nosocomial HiRECC analyzed in our study carried such molecular markers as IS*16* and the *esp* gene together with the *intA* gene characteristic for ICE*Efm*[41,42]. Another pathogenicity factor, the *fms19* gene of the pilin gene cluster 4 (PGC4) showed increased prevalence among these isolates. Similar observation was made for a representative collection of 433 *E. faecium* isolates from various sources [43].

In a study that followed enterococci in a continuum from hospital and retirement home wastewaters through a WWTP to a river, a gradual decrease in the proportion of *E. faecium* isolates representing hospital-associated STs was observed, likely due to the “dilution” of HiRECC representatives by more diverse and less drug-resistant strains originating from the community [28]. Because in our study only selected representatives of obtained isolates were investigated, it is not possible to determine precise tendencies in their frequency from the WWTP influent to effluent and water, however, resistant enterococci, both HiRECC and non-HiRECC, are clearly able to survive the treatment process, what results in their release into marine and river waters. Moreover, using an additional typing method, MLVA, we were able to find isolates with the same characteristics in the effluent of WWTP and its marine outfall, suggesting ability of such clones to survive in the environment for at least some time. It seems plausible that hospital resistant clones accumulate several determinants that may promote their survival not only in the hospital settings but also in the environment. Such features would include resistance to disinfectants and ultraviolet light, additional metabolic pathways and ability to form biofilms. Moreover, determinants of some of the mentioned adaptation factors are found co-resident in plasmids harboring resistance genes, suggesting co-selection. For example, pLG1-type plasmids of *E. faecium* carry not only resistance determinants against aminoglycosides, glycopeptides and macrolides but also pili genes and carbon uptake-utilization genes [38,39]. Pheromone-responsive plasmids of *E. faecalis* apart from resistance to aminoglycosides, glycopeptides, penicillins and macrolides may encode bacteriocins, additional pili and UV-resistance determinants [40]. Thus, nosocomial HiRECCs, released by WWTPs, are equipped in whole sets of genes with various adaptive benefits and often located within MGE. It was also demonstrated that MGEs can be transferred in a WWTP [44], further underling the importance of threat posed by such organisms. There is a selection pressure for survival of resistant strains and their tranconjugants due to release of antibiotics to the environment, especially in the hospital effluents [45], and even sub-inhibitory concentrations may be effective in selection for resistance [46].

## Conclusions

This study describes the species composition, resistance profiles and clonal relationships of enterococci isolated from WWTPs located in Gdansk, their marine outfalls to the Gulf of Gdansk and from Vistula River. It was observed that these WWTPs release enterococcal strains resistant to various antimicrobial compounds to the recipient water environment. Molecular analyses revealed the presence of clones associated with nosocomial HiRECCs, and, in the case of *E. faecium* isolates of lineages 17, 18 and 78 of former CC17 abundant in resistance determinants, MGE genes and pathogenicity factors. The presence of HiRECCs in wastewaters and marine/river environment highlights the need for further detailed analyses to better understand the survival and spread of drug-resistant strains in water ecosystems, and to elucidate the ways to curb such dissemination.

## Methods

### Acquisition of samples and isolation of enterococci

Once a month in April, June, August and October in 2011 of the flow proportioned composited samples of wastewater were taken from two local WWTPs: Gdansk-Wschod from influent (W-INF), effluent (W-EFF), and wastewater from activated sludge bioreactor (W-BR) and from Gdynia-Debogorze effluent (D-EFF). The two WWTPs treat sewage from approximately 920 000 inhabitants (for details see Figure 1). In the same period of time and with the same frequency marine outfalls of those WWTPs (W-MOut and D-MOut, respectively) as well as Vistula River mouth were also sampled for isolation of enterococci. Marine samples were taken at the depth of approximately one meter below the surface once a month in April, June, August and October of 2011. Location of sampling points and their characteristics are provided in Figure 1. Enterococci were detected using the membrane filtration method. To this end, appropriate dilutions of analyzed samples were filtered in duplicate through 0.45 μm cellulose-acetate filters (EMD Millipore Corporation, Billerica, MA, USA), which were then placed on Enterococcus selective agar (Merck, Darmstadt, Germany) and incubated at 37°C for 48 h (ISO 7899–2:2000). Dark red or maroon colonies were considered presumptive enterococci. For further investigations, representative isolates were taken from membranes presenting from 20 to 50 typical colonies and stored in nutrient broth supplemented with 15% glycerol at −80°C.

### Species identification, DNA isolation, susceptibility testing and statistical analysis

The species identification and drug susceptibility of presumptive enterococci were determined by the Phoenix™ Automated Microbiology System (BD, New Jersey, USA) according to the manufacturer’s instructions. Since it is not possible to distinguish *E. casseliflavus* and *E. gallinarum* using this system, the classification: *E. casseliflavus/gallinarum* was used. The susceptibility tests, based on microdilution, were carried out for 11 antimicrobial agents: gentamicin (GM), streptomycin (ST), ampicillin (AM), daptomycin (DAP), vancomycin (VA), teicoplanin (TEC), linezolid (LZD), fluoroquinolones (ciprofloxacin - CIP, levofloxacin - LVX), tetracycline (TE), erythromycin (E) and quinupristin-dalfopristin (SYN) (*E. faecium* only). Obtained minimal inhibitory concentrations (MIC) were evaluated according to the guidelines of Clinical and Laboratory Standards Institute [47]. Isolates were defined as multidrug-resistant (MDR) when they showed resistance to three or more compounds tested [48]. Bacterial DNA was purified using the Genomic DNA Prep Plus kit following the manufacturer’s instructions (A&A Biotechnology, Gdynia, Poland). Differences in distributions were evaluated using the χ2 test, with the p values < 0.05 considered significant.

### MLST and MLVA of *E. faecium*

MLST for *E. faecium* was performed by sequencing of seven house-keeping genes as described by Homan *et al*. [49]. The allele numbers and STs were assigned with the use of Internet database http://efaecium.mlst.net/ (5^th^ July 2013, date last accessed). Novel alleles and allelic profiles were submitted to the database. MLST data were analyzed with Sequence Type Analysis and Recombinational Tests (START) software [50] and the eBURST analysis [51] was used to elucidate the relationships of STs with known CCs, using the comparative eBURST option available at http://eburst.mlst.net/v3/enter_data/comparative/ (5^th^ July 2013, date last accessed). Further inclusion of *E. faecium* STs into specific CCs and lineages was performed following other studies [9,24]. MLVA was performed as described by others [52].

### Detection and analysis of genes associated with virulence, antimicrobial resistance and MGE in *E. faecium*

The full list of primers used for gene detection and sequencing is provided in Additional file 1: Table S1. PCR-based detection of IS*16,* virulence-associated genes (*fms21*, *fms17, fms5* and *fms19,* representing each of four pilin gene clusters in *E. faecium,* and the *esp*_Efm_ gene), genes determining resistance to tetracycline {*tet*(M)*, tet*(O)*, tet*(L)*, tet*(S)}, HLGR (*aac(6′)-Ie-aph(2″*), HLSR (*ant(6′)-Ia*) and genes specific for MGE, such as *intA* of ICE*Efm1*, *int* of Tn*916* transposon, *tndX* of Tn*5397* transposon*,* enterococcal plasmid replication genes *rep1*_pIP501_*, rep2*_pRE25_*, rep17*_pRUM_*, rep18*_pEF418_*, rep*_pMG1_, *rep*_pLG1_ and TAS genes *axe-txe,* ω*-ε-ζ, relBE* was performed as described in other studies [13,41,53-59], following the conditions described herein. Previously characterized clinical isolates of *Streptococcus agalactiae, E. faecium* and *E. faecalis* harbouring pilin genes, IS*16*, *esp*_Efm_*, intA* of ICE*Efm1*, *int* of Tn*916*, *tndX* of Tn*5397, tet*(M)*, tet*(O)*, tet*(L)*, tet*(S)*, aac(6′)-Ie-aph(2″*), *ant(6′)-Ia*, *rep1*_pIP501_*, rep2*_pRE25_, ω*-ε-ζ*[13,60,61] served as positive controls. In cases when positive controls were not available (*rep18*_pEF418_*, rep*_pMG1_, *rep*_pLG1_, *axe-txe, relBE*), a few randomly selected PCR products were verified by sequencing and served further as controls. Ampicillin-resistance determinant, the *pbp5* gene was analysed by sequencing of the part encoding C-terminal transpeptidase domain in PBP5 [62]. New sequence variants, *pbp5-24* to *pbp5-29* alleles were submitted to the GenBank (acc. numbers KC594860-KC594865, respectively). The quinolone-determining regions (QRDR) in the *gyrA* and *parC* genes were sequenced and analysed as described by others [63].

## Competing interest

The authors declare that they have no competing interest.

## Authors’ contributions

ES participated in the study design, performed experimental work and participated in the manuscript preparation; AL designed the study, performed experimental work and participated in the manuscript preparation. Both authors read and approved the final manuscript.

## Supplementary Material

Additional file 1: Table S1 Primers used in the study for PCR and sequencing. Click here for file
