# Supplementary material for: Drug-resistant and hospital-associated Enterococcus faecium from wastewater, riverine estuary and anthropogenically impacted marine catchment basin
Source: BMC Microbiol. 2014 Mar 14;14:66. doi: 10.1186/1471-2180-14-66 (PMC4004213; doi:10.1186/1471-2180-14-66)
Supplement: Additional file 1: Table S1 — Primers used in the study for PCR and sequencing. [file 1471-2180-14-66-S1.doc]

Supplementary table 1. Primers used in the study for PCR and sequencing.

| Target gene | Sequence5’->3’ | Reference |
| --- | --- | --- |
| IS*16* | CATGTTCCACGAACCAGAG | ‎51 |
|  | TCAAAAAGTGGGCTTGGC | ‎51 |
| *fms21* | CTTATTGGAATGTTAGGAATCAT | ‎24 |
|  | TCAGTAGCAGTCAGCTTTCC | ‎24 |
| *fms17* | ATGAAAATGATGGCTTGGCT | ‎24 |
|  | GATGATGACCTCGATTCTC | ‎24 |
| *fms5* | ATGTTTACCGCAGAAGCAAC | ‎24 |
|  | ACTTGTATCCGTTGGCTGTT | ‎24 |
| *fms19* | GTGTGGAAGACGCACAAAGA | ‎24 |
|  | GGGACTTTATCCCCATCTGC | ‎24 |
| *esp*_Efm_ | AGATTTCATCTTTGATTCTTGG | ‎66 |
|  | AATTGATTCTTTAGCATCTGG | ‎66 |
| *tet*(M) | AGTTTTAGCTCATGTTGATG | ‎67 |
|  | TCCGACTATTTAGACGACGG | ‎67 |
| *tet*(O) | AGCGTCAAAGGGGAATCACTATCC | ‎68 |
|  | CGGCGGGGTTGGCAAATA | ‎68 |
| *tet*(L) | ATAAATTGTTTCGGGTCGGTAAT | ‎68 |
|  | AACCAGCCAACTAATGACAAGT | ‎68 |
| *tet*(S) | CCATTGGGAAAACATTGAATATTGC | ‎18 |
|  | GCTCTGCCTTATCTATCTGG | ‎18 |
| *aac(6′)-Ie-aph(2″*) | GAGCAATAAGGGCATACCAAA | ‎69 |
|  | GTTCCTATTTCTTCTTCACTATCTTCA | ‎69 |
| *ant(6′)-Ia* | GCCCTTGGAAGAGTTAGATAATT | ‎69 |
|  | CGGCACAATCCTTTAATAACA | ‎69 |
| *intA* | GAGCCAATTCAGGATGTCG | ‎18 |
|  | GAAAGAAGGATACGGGAAGGT | ‎18 |
| *int*_Tn_*_916_* | GCGTGATTGTATCTCACT | ‎67 |
|  | GACGCTCCTGTTGCTTCT | ‎67 |
| *tndX* | ATGATGGGTTGGACAAAGA | ‎70 |
|  | CTTTGCTCGATAGGCTCTA | ‎70 |
| *rep1*_pIP501_ | TCGCTCAATCACTACCAAGC | ‎71 |
|  | CTTGAACGAGTAAAGCCCTT | ‎71 |
| *rep2*_pRE25_ | GAGAACCATCAAGGCGAAAT | ‎71 |
|  | ACCAGAATAAGCACTACGTACAATCT | ‎71 |
| *rep17*_pRUM_ | TACTAACTGTTGGTAATTCGTTAAAT | ‎71 |
|  | ATCAAGGACTCAACCGTAATT | ‎71 |
| *rep18*_pEF418_ | ACACCAGTCGAAATGAATTT | ‎71 |
|  | AGGAATATCAAGTAATTCATGAAAGT | ‎71 |
| *rep*_pMG1_ | GTATTAACACACTGGACTC | ‎71 |
|  | TCAGTGTAGGCAATAACCC | ‎71 |
| *rep*_pLG1_ | GAAAATGATATCTACTTACTCG | ‎73 |
|  | TTACATAGACAAAAATCAGGT | ‎73 |
| *axe-txe* | CTGACCCTTTCCTTACTTCCG | ‎72 |
|  | GGGTGAAAGGAATGGAAGCAG | ‎72 |
| ω*-ε-ζ* | GTGGTTTAGGTGGCTGCAAG | ‎72 |
|  | TTAACGAATTATCGGCAAGC | ‎72 |
| *relBE* | CAGAGAATGCGTTTGACCG | ‎72 |
|  | GGTGTAACTCCTTCTGAAGCG | ‎72 |
| *pbp5* | CGGGATCTCACAAGAAGAT | ‎75 |
|  | TTATTGATAATTTTGGTT | ‎75 |
| *gyrA* | CGGGATGAACGAATTGGGTGTGA | ‎76 |
|  | AATTTTACTCATACGTGCTTCGG | ‎76 |
| *parC* | TTCCCGTGCATTTCGATCAGTACTTC | ‎76 |
|  | CGTATGACAAAGGATTCGGTAAATC | ‎76 |
